# Supplementary material for: Barrier properties of Nup98 FG phases ruled by FG motif identity and inter-FG spacer length
Source: Nat Commun. 2023 Feb 10;14:747. doi: 10.1038/s41467-023-36331-4 (PMC9918544; doi:10.1038/s41467-023-36331-4)
Supplement: Supplementary file 2 — Reporting Summary [file 41467_2023_36331_MOESM2_ESM.pdf]

nature portfolio

Corresponding author(s): Dirk Görlich

Last updated by author(s): Jan 8, 2023

Reporting Summary

Nature Portfolio wishes to improve the reproducibility of the work that we publish. This form provides structure for consistency and transparency in reporting. For further information on Nature Portfolio policies, see our Editorial Policies and the Editorial Policy Checklist.

Statistics

For all statistical analyses, confirm that the following items are present in the figure legend, table legend, main text, or Methods section.

n/a	Confirmed	
☐	☒	The exact sample size (*n*) for each experimental group/condition, given as a discrete number and unit of measurement
☐	☒	A statement on whether measurements were taken from distinct samples or whether the same sample was measured repeatedly
☒	☐	The statistical test(s) used AND whether they are one- or two-sided<br>*Only common tests should be described solely by name; describe more complex techniques in the Methods section.*
☒	☐	A description of all covariates tested
☒	☐	A description of any assumptions or corrections, such as tests of normality and adjustment for multiple comparisons
☐	☒	A full description of the statistical parameters including central tendency (e.g. means) or other basic estimates (e.g. regression coefficient) AND variation (e.g. standard deviation) or associated estimates of uncertainty (e.g. confidence intervals)
☒	☐	For null hypothesis testing, the test statistic (e.g. *F*, *t*, *r*) with confidence intervals, effect sizes, degrees of freedom and *P* value noted<br>*Give P values as exact values whenever suitable.*
☒	☐	For Bayesian analysis, information on the choice of priors and Markov chain Monte Carlo settings
☒	☐	For hierarchical and complex designs, identification of the appropriate level for tests and full reporting of outcomes
☒	☐	Estimates of effect sizes (e.g. Cohen's *d*, Pearson's *r*), indicating how they were calculated

Our web collection on statistics for biologists contains articles on many of the points above.

Software and code

Policy information about availability of computer code

Data collection	Leica Application Suite X 3.3.0, Matlab R2020a. All software used is commercially available.
Data analysis	- Microsoft Excel version 16.42, Leica Application Suite X 3.3.0, Matlab R2020a. The above software is commercially available.<br>- FIJI 2.9.0 (Schindelin et al., 2012 Fiji: an open-source platform for biological-image analysis. Nat Methods 9, 676-682)<br>Custom Matlab scripts for ODT reconstruction can be found on: https://github.com/OpticalDiffractionTomography

For manuscripts utilizing custom algorithms or software that are central to the research but not yet described in published literature, software must be made available to editors and reviewers. We strongly encourage code deposition in a community repository (e.g. GitHub). See the Nature Portfolio guidelines for submitting code & software for further information.

Data

Policy information about availability of data

All manuscripts must include a data availability statement. This statement should provide the following information, where applicable:

- Accession codes, unique identifiers, or web links for publicly available datasets
- A description of any restrictions on data availability
- For clinical datasets or third party data, please ensure that the statement adheres to our policy

The data generated in this study are provided in the Supplementary Information/Source Data file.

Human research participants

Policy information about studies involving human research participants and Sex and Gender in Research.

Reporting on sex and gender	This is not applicable to our study as we do not have human research participants.
Population characteristics	This is not applicable to our study as we do not have human research participants.
Recruitment	This is not applicable to our study as we do not have human research participants.
Ethics oversight	This is not applicable to our study as we do not have human research participants.

Note that full information on the approval of the study protocol must also be provided in the manuscript.

Field-specific reporting

Please select the one below that is the best fit for your research. If you are not sure, read the appropriate sections before making your selection.

☒ Life sciences ☐ Behavioural & social sciences ☐ Ecological, evolutionary & environmental sciences

For a reference copy of the document with all sections, see nature.com/documents/nr-reporting-summary-flat.pdf

Life sciences study design

All studies must disclose on these points even when the disclosure is negative.

Sample size	Sample sizes were not predetermined by statistical methods, but determined by the variability of the replicates. E.g., in FG particle permeation assays, S.D. were typically <10% between individual particles (as shown in the images) and between replicates as reported in this study and previously (Frey et al., 2018 Surface properties determining passage rates of proteins through nuclear pores. Cell, 174(1), 202-217.e9 and Ng et al., 2021 Recapitulation of selective nuclear import and export with a perfectly repeated 12mer GLFG peptide. Nat Commun, 12(1), 4047), and thus the lower limit of the sample size was set to 3. Note that the S.D. is minute as compared to the observed effects (e.g., 50 000-fold differences between NTF2 and mCherry partition coefficients and up to 600-fold differences between FG domain variants). In phase separation tests for the determination of saturation concentrations, S.D. between replicates were typically <20% in this study and previously (Najbauer et al., 2022 Atomic resolution dynamics of cohesive interactions in phase-separated Nup98 FG domains. Nat Commun, 13(1)) and were relatively small if saturation concentrations were presented in the natural logarithm scale. Therefore the lower limit of the sample size was set to 2. Note that the overall trends are clear and reproducible (e.g., R-squared value in Fig.2c is better than 0.99), suggesting that the sample size is sufficient. In ODT measurements, the range of differences between variants is within a factor of two, therefore the sample size was set to 10 (FG particles) to allow for a clear trend.
Data exclusions	No data were excluded.
Replication	Replications were performed on independent samples. Numbers of replicates were stated in the figure legends/Methods. Attempts at replication were successful.
Randomization	Our samples (FG domain variants) were not taken from a preexisting distribution but were designed and produced for specific purposes and specific measurements. Therefore randomization is not applicatable, with respect to the experimental design. However, the allocations of FG particles/ HeLa cells into wells of plates for tests were random.
Blinding	Phase separation tests: Investigators were not blinded during the analyses because this is a simple one-step experiment without much room for mishandling/ false-positives/false-negatives, and phase separation at our assay volume was clearly visible by naked eyes of anyone (as indicated by sample turbidity in a clearly visible volume) immediately after the sample was brought to the experimental condition and thus the influence of subjectivity was not significant enough to require blinding. In the analyses of variants with varying spacer length or FG motifs, the differences between the variants were well-recognizable by naked eyes and again, the influence of subjectivity was not significant enough to require blinding.<br>FG particle permeation assays and confocal laser scanning microscopy: Investigators were not blinded during the analyses, because otherwise the investigators would have been unable to apply the relevant measurement settings (e.g. optimal laser power) to specific samples. These assays are also simple set-ups without much room for mishandling. Moreover, there are well-recognizable differences in the readouts from our samples (e.g., GLFG52x10 versus GLFG52x15 or GLFG52x12 versus GFLG52x12) and thus the influence of subjectivity is not significant enough to require blinding. The authors declared that the non-imaged areas showed no noticeable difference compared to the imaged areas of the same sample.<br>ODT and NPC-staining tests : Investigators were blinded during the analyses.

Reporting for specific materials, systems and methods

We require information from authors about some types of materials, experimental systems and methods used in many studies. Here, indicate whether each material, system or method listed is relevant to your study. If you are not sure if a list item applies to your research, read the appropriate section before selecting a response.

Materials & experimental systems

n/a	Involved in the study
☒	☐ Antibodies
☐	☒ Eukaryotic cell lines
☒	☐ Palaeontology and archaeology
☒	☐ Animals and other organisms
☒	☐ Clinical data
☒	☐ Dual use research of concern

Methods

n/a	Involved in the study
☒	☐ ChIP-seq
☒	☐ Flow cytometry
☒	☐ MRI-based neuroimaging

Eukaryotic cell lines

Policy information about cell lines and Sex and Gender in Research

Cell line source(s)	HeLa-Kyoto (ECACC)
Authentication	ECACC; RRID:CVCL_1922
Mycoplasma contamination	Tested negative
Commonly misidentified lines (See ICLAC register)	None
